# Heme orientation modulates histidine dissociation and ligand binding kinetics in the hexacoordinated human neuroglobin

**DOI:** 10.1007/s00775-012-0956-2

**Published:** 2012-11-08

**Authors:** Anthony Bocahut, Valérie Derrien, Sophie Bernad, Pierre Sebban, Sophie Sacquin-Mora, Eric Guittet, Ewen Lescop

**Affiliations:** 1 Laboratoire de Chimie Physique, UMR 8000, CNRS, Université Paris-Sud 11, Bât. 350, 91405 Orsay, France; 2 Laboratoire de Biochimie Théorique, UPR 9080, Institut de Biologie Physico-Chimique, CNRS, 13 rue Pierre et Marie Curie, 75005 Paris, France; 3 Centre de Recherche de Gif, Institut de Chimie des Substances Naturelles, CNRS, 1 avenue de la Terrasse, 91190 Gif-sur-Yvette, France; 4 Université des Sciences et des Technologies de Hanoi, 18 Hoang Quoc Viet, Cau Giay, Hanoi, Vietnam

**Keywords:** NMR, Neuroglobin, Molecular dynamics, Heme orientation, Disulfide bridge

## Abstract

Neuroglobin (Ngb) is a globin present in the brain and retina of mammals. This hexacoordinated hemoprotein binds small diatomic molecules, albeit with lower affinity compared with other globins. Another distinctive feature of most mammalian Ngb is their ability to form an internal disulfide bridge that increases ligand affinity. As often seen for prosthetic heme *b* containing proteins, human Ngb exhibits heme heterogeneity with two alternative heme orientations within the heme pocket. To date, no details are available on the impact of heme orientation on the binding properties of human Ngb and its interplay with the cysteine oxidation state. In this work, we used ^1^H NMR spectroscopy to probe the cyanide binding properties of different Ngb species in solution, including wild-type Ngb and the single (C120S) and triple (C46G/C55S/C120S) mutants. We demonstrate that in the disulfide-containing wild-type protein cyanide ligation is fivefold faster for one of the two heme orientations (the A isomer) compared with the other isomer, which is attributed to the lower stability of the distal His64–iron bond and reduced steric hindrance at the bottom of the cavity for heme sliding in the A conformer. We also attribute the slower cyanide reactivity in the absence of a disulfide bridge to the tighter histidine–iron bond. More generally, enhanced internal mobility in the CD loop bearing the disulfide bridge hinders access of the ligand to heme iron by stabilizing the histidine–iron bond. The functional impact of heme disorder and cysteine oxidation state on the properties of the Ngb ligand is discussed.

## Introduction

**Fig. 1:**
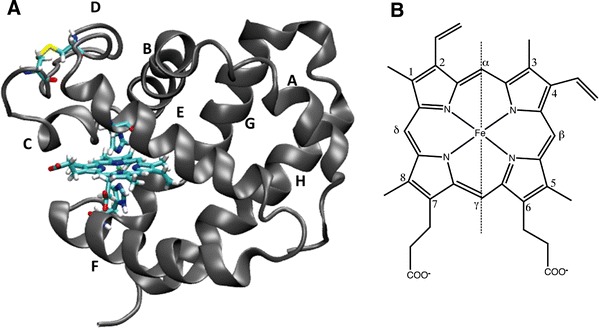
**a** Structure of human neuroglobin (Ngb) with the Cys46-Cys55 disulfide bridge formed obtained by molecular dynamics simulations [27]. The prosthetic group (heme), the two distal (His64) and proximal (His96) histidines, and the two cysteines (Cys46 and Cys55), located on the CD loop, are represented as licorice (VMD drawing method). **b** Structure of the heme molecule. The carbon atoms are numbered according to the Fischer numeration and the *α*–*γ* *meso* axis is shown as a *dashed line*

Neuroglobin (Ngb) is a small protein discovered 10 years ago in the nervous system of vertebrates [1]. Ngb is a new member of the large globin family and in contrast to other globins that are often expressed in all organs, Ngb is mostly present in brain, retina, and other nerve tissues [2–5]. The globin family possesses a characteristic eight *α*-helix fold (see Fig. 1a) and heme *b* as a prosthetic group which is able to bind small molecules at its central iron atom [6–11].

Similarly to other globins, small ligands (O_2_, NO, CO) can bind deoxyNgb (Fe^2+^), whereas the metNgb (Fe^3+^) form is able to bind small ligands such as CN^−^, NO, and N_3_^−^ [12]. However, the low affinity for O_2_ precludes a role as an O_2_ carrier [3–5, 13]. The function of Ngb has not been fully resolved, and different roles for Ngb have been proposed in the past [2–5, 13–17]. For example, its potential interaction with the NO radical led to a neuroprotective role in the case of oxidative stress in cells being proposed [18–20]. Furthermore the expression of Ngb increases during hypoxia, which may reveal the action of Ngb as a radical scavenger [2–5].

Despite the low (25 %) sequence homology between Ngb and other members of the globin family, Ngb shares the highly conserved amino acid positions characteristic of the globin fold [21–23]. Not surprisingly, the crystal structure of Ngb [22, 23] revealed strong structural similarities between Ngb and other globins. Similarly to a few other globins such as cytoglobin and nonsymbiotic plant hemoglobins, Ngb possesses two key histidine residues in the iron coordination sphere, including the distal His64 (E7) and the proximal His96 (F8) that close the coordination sphere of the heme iron atom, resulting in a hexacoordinated iron even in the absence of an external ligand. This situation contrasts with that in most globins, for which only the proximal histidine is coordinated to the iron and the distal pocket is directly available to host an external diatomic ligand without the requirement for conformational change. It has been proposed that in Ngb the distal histidine has first to be dissociated to make the distal pocket accessible to the incoming ligand [24, 25]. A recent experimental study on mouse Ngb [26] and a theoretical study on human Ngb [27] also suggested that His64–iron bond breaking is accompanied by heme sliding instead of histidine side chain rotation.

In the case of globins, protoheme IX is not directly linked to the peptidic backbone. Owing to the lack of true twofold symmetry, heme *b* often adopts two stable conformations, A and B, in the heme pocket; they differ by 180° rotation around the *α*–*γ* *meso* axis (see the vertical dashed line in Fig. 1a). For example, the M1 methyl (and V2 vinyl) group in the A form occupies a spatial position similar to that of the vinyl V4 (and M3 methyl, respectively) group in the B form, and vice versa. The ratio of A and B populations is highly sensitive to the composition of the heme pocket and thus depends on protein sequences and species. For example, the crystal structures of mouse Ngb (Protein Data Bank entries 1Q1F and 2VRY) contain two heme molecules refined as 70 and 30 % occupancy and labeled as A and B, respectively [23]. The two stable orientations of heme are also reflected in the ^1^H NMR spectra of mouse and human Ngb, with a major (70 %) B conformation and a minor (30 %) A conformation [28–30]. As a source of confusion [31], different conventions were used for the NMR and crystallographic studies. In this article, we use the same nomenclature as previous NMR studies of Ngb. Following this naming convention, the groups contacting the Phe42 side chain are M8/M1 in the B conformer and M5/V4 in the A conformer.

Another unique characteristic of human Ngb is the existence in the CD–D region of two cysteine residues, Cys46 (CD7) and Cys55 (D5). The first experimental evidence for the existence in human Ngb of an internal disulfide bridge involving these cysteine residues was reported in [32, 33]. Remarkably, an increase in O_2_ affinity [32, 33] and CO rebinding rate [34] was observed upon formation of the bridge, suggesting that cysteines in human Ngb may act as a strong regulator of O_2_ binding and may be used as redox sensors. Unfortunately, no high-resolution structure is yet available for human wild-type (WT) Ngb with the internal disulfide bond. However, the crystal structures of mouse Ngb and the cysteine-depleted human Ngb, both lacking the crucial cysteines, as well as molecular dynamics (MD) simulations [27, 35] suggest the requirement for a local rearrangement in the CD–D region located between the two *α*-helices C and D to accommodate the formation of the internal disulfide bridge. The cysteine redox-dependent ligand binding properties of Ngb were then proposed to be associated with the large conformational change of the CD loop required to bring the thiol groups in close proximity [27, 35]. Our understanding of the conformational change and of the precise role of the cysteine redox state in human Ngb function is still limited, emphasizing the need for better characterization of Ngb with oxidized (WT_ox_) or reduced (WT_red_) cysteines. Mammalian Ngb sequences are highly conserved (more than 90 % sequence identity). However, in rodent Ngb, a conserved glycine is systematically found at the position equivalent to Cys46 in the human Ngb sequence and represents the sole substitution in the otherwise strictly conserved CD loop. Hence, despite bearing the conserved cysteines at positions 55 and 120 (in the human sequence), rodent Ngb does not have the ability to create an internal disulfide bridge, which may reflect possible functional differences between rodents and other mammals.

Similarly to many hemoproteins, the Ngb protein is capable of existing in several stable states characterized by the oxidation state of the heme iron, the heme orientation, and the nature of the sixth ligand of the iron (distal histidine or external small ligand). The possibility to form an internal disulfide bridge is unique to nonrodent mammalian Ngb compared with other globins and further increases the structural diversity. A growing body of evidence has been gathered in the last decade demonstrating the profound influence of the conformation of Ngb on its ligand binding properties [32–34], but a systematic study was still lacking. In this work, we explored the spectroscopic properties of the various human Ngb forms by ^1^H NMR spectroscopy. The excellent spectral dispersion due to the paramagnetic high-spin ferric ion (Fe^3+^) and its ability to directly monitor various species in solution that cannot be purified to homogeneity, such as the slowly interconverting heme orientations, establish NMR spectroscopy as a convenient tool to assess the influence of the disulfide bridge and/or the heme orientation on the binding kinetics and thermodynamics of Ngb with the small-ligand cyanide anion. The Fe^3+^–CN^−^ complex is believed to be isostructural and isoelectronic to the physiologically relevant Fe^2+^–CO complex and its very slow binding permits time-resolved NMR investigations, which are impossible in the case of the (fast) binding of CO or O_2_. Furthermore, we provide new MD trajectories highlighting the role of dynamics in the ligation mechanism. Beyond the specific study of Ngb, the understanding of the influence of heme orientation on protein reactivity will extend our knowledge of hemoproteins.

## Materials and methods

### Cloning, expression and purification of recombinant Ngb

Recombinant human WT Ngb was cloned into the expression vector pET15b and overexpressed in *Escherichia coli* BL21 (DE3). The sequence of Ngb encompassed the six His-tag at the N-terminal extremity followed by Leu-Val-Pro-Arg-Gly-Ser. The overexpression and the preparation of a crude Ngb extract was performed as described by Dewilde et al. [36]. The proteins were purified on a nickel nitrilotriacetic acid resin column using an equilibrium buffer [50 mM tris(hydroxymethyl)aminomethane (Tris)–HCl pH 8.0, 8 mM imidazole] and an eluent buffer (50 mM Tris–HCl pH 8.0, 150 mM imidazole). The His-tag of the different samples was cleaved using a thrombin kit (Sigma) and concentrated in 20 mM Tris–HCl pH 7.4. Ngb samples were stored at 193 K prior to use. For Ngb mutants, the QuikChange site-directed mutagenesis method was used to generate the C120S single mutant and the C46G/C55S/C120S triple mutant starting from human Ngb as a template.

### NMR spectroscopy

Ngb samples were prepared in 20 mM Tris–HCl buffer at pH 7.4 at 100 μM protein concentration and 90/10 % H_2_O/D_2_O. NMR experiments were performed at 298 K using a Bruker AVANCE II 600 MHz spectrometer equipped with a TCI cryoprobe. Data processing and analysis were performed using NMRpipe [37] and CCPNMR [38] software programs, respectively. The CN^−^ binding experiment was initiated by the addition of 500 μM KCN to the protein solution and followed by a series of 1D ^1^H spectra. Solvent peak suppression was achieved using a water on-resonance 1-s presaturation. The spectral width was set to 80 ppm. One 1D spectrum was typically collected every 20 min (1,024 transients) to follow spectral changes. The KCN binding experiment was performed on the WT, C120S, and triple-mutant proteins. For the WT and C120S proteins, the internal disulfide bridge was either oxidized or reduced prior to KCN addition. The reduction of the disulfide bridge was obtained by adding 2 mM dithiothreitol (DTT) to the protein solution and the excess DTT was removed by a three-step dilution/concentration method using Vivaspin centrifugal concentrators (Sartorius Stedim, *M*_r_ = 10,000, 3,000g). The dilution step was achieved using the 20 mM Tris–HCl (pH 7.4) buffer.

### Classical MD

MD simulations were performed with the Gromacs software package [39–41] using the OPLS all-atom force field [42]. The starting coordinates employed for the simulations were taken from the experimental X-ray structure of the cysteine-depleted human Ngb at 1.95-Å resolution (Protein Data Bank entry 1OJ6, B chain) [22]. The charges and parameters for the prosthetic group were previously described by Bocahut et al. [27]. The protein was solvated in a 78-Å cubic box, using periodic boundary conditions, with explicit single-point charge [43] water molecules; six Na^+^ ions were added to neutralize the system, which contained a total of around 48,000 atoms. The dynamics was performed at 1 atm and 300 K, maintained with the barostat and thermostat of Berendsen et al. [44]. Long-range electrostatic interactions were treated using the particle mesh Ewald method [45], with a grid spacing of 0.12 nm and a nonbond pair list cutoff of 9.0 Å with updating of the pair list every five steps. We chose a time step of 2 fs by constraining bond lengths involving hydrogen atoms with the LINCS algorithm [46]. The solvent was first relaxed by an energy minimization followed by a 100-ps equilibration step under restraint, and then heated slowly until a temperature of 300 K was reached for the system; 12-ns production runs were eventually performed, from which the last 10 ns was kept for analysis. In particular, the MD trajectory was investigated using principal component analysis [46–49] on the first eight normal modes of the protein in order to retrieve the most significant fluctuations occurring along the collective modes of motion of Ngb.

## Results

### NMR characterization of the oxidized WT Ngb (WT_ox_)

**Fig. 2:**
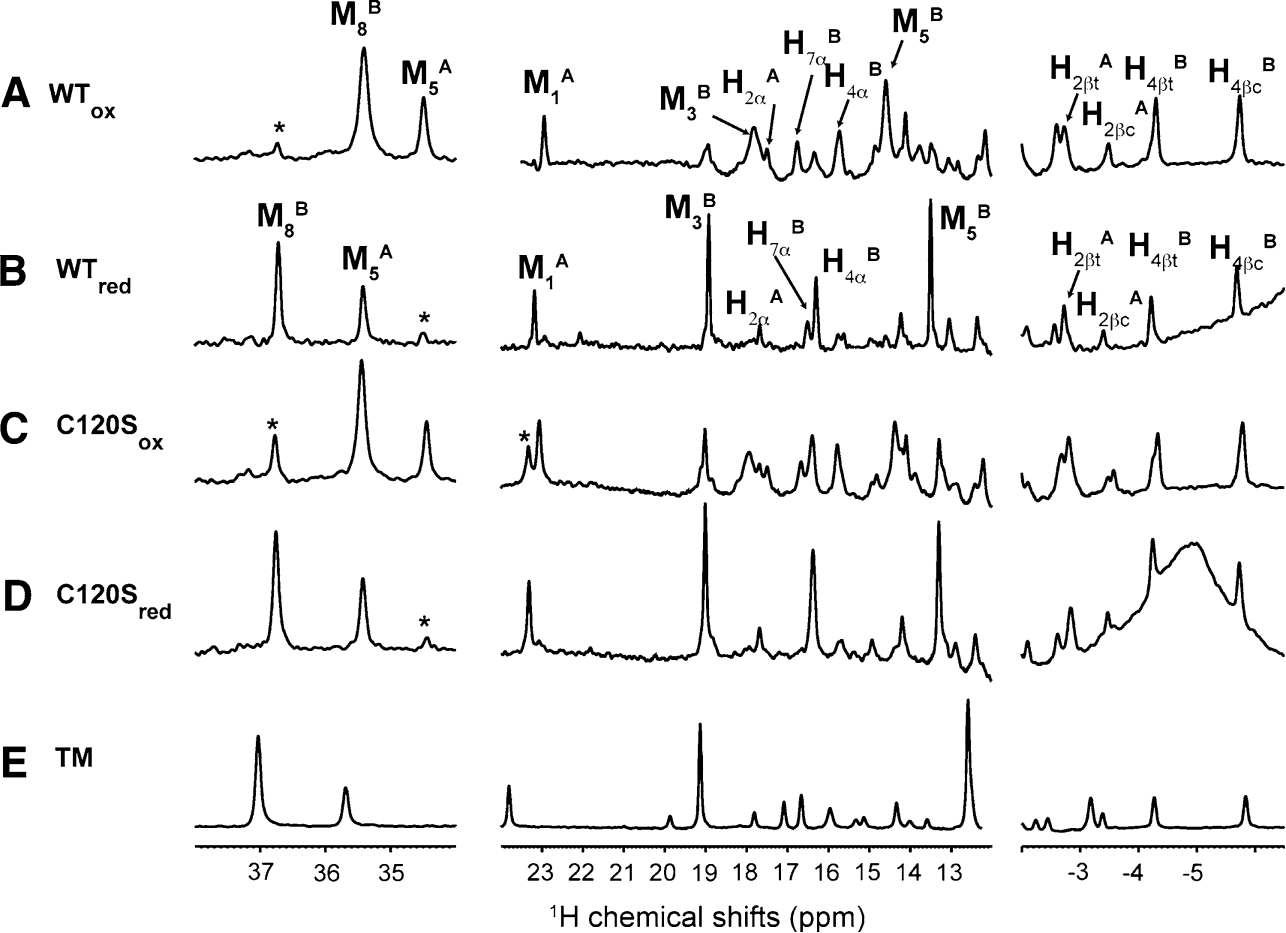
One-dimensional ^1^H NMR spectra of the Ngb protein. Spectra *A* and *B* were collected on the wild-type (WT) Ngb protein without dithiothreitol treatment (*WT*_*ox*_) or after dithiothreitol treatment (*WT*_*red*_), respectively. Spectra *C* and *D* are the spectra of oxidized and reduced C120S Ngb. Spectrum *E* is the 1D spectrum of C46G/C55S/C120S triple-mutant (*TM*) Ngb. All NMR spectra were collected at 298 K in 20 mM tris(hydroxymethyl)aminomethane–HCl buffer (pH 7.4) at 100 μM protein concentration using a 600-MHz NMR spectrometer. The assigned heme atoms are labeled for WT Ngb in the WT_ox_ and WT_red_ states. Peaks labeled with a *star* correspond to incomplete reduction or oxidation of the protein

WT Ngb was prepared in the absence of reducing agent during cell lysis and purification steps. Figure 2, spectrum A shows the ^1^H NMR spectrum collected at 298 K immediately after the last purification step. The low-field region (30–40 ppm) of ^1^H spectra of hemoproteins is of high interest since it usually contains several lines corresponding to heme M5 or M8 methyls, which facilitates the evaluation of sample homogeneity. Two major signals were visible at 35.4 and 34.5 ppm that were previously assigned to the M8B and M5A methyls, respectively [30]. The letters A and B correspond to the different orientations of the heme moiety within the pocket. Owing to this pseudosymmetry, the M5 methyl group in conformer A occupies a position similar to that of the M8 methyl group in the B conformer, and thus both exhibit similar paramagnetic shifts. As previously noted [30], the B conformer was preferentially populated with respect to the A conformer with a population ratio close to 2:1 as estimated from peak volumes. Several additional weak signals were also observed (marked with a star in Fig. 2, spectrum A) and revealed incomplete oxidation of the cysteines.

### NMR characterization of the DTT-reduced WT Ngb (WT_red_)

To characterize the properties of Ngb with reduced cysteines, we incubated the Ngb sample with DTT and extensively washed the protein solution to remove oxidized or reduced DTT prior to NMR measurement. The reduction of the disulfide bridge was also checked by mass spectrometry. We observed a 2.4-Da increase in mass upon reduction by DTT, in agreement with the formation a single disulfide bridge in Ngb before DTT treatment and with the two additional hydrogens in reduced cysteines. This analysis was also consistent with a highly predominant monomeric form of the protein before and after DTT treatment. The ^1^H NMR spectrum of Ngb subjected to this treatment is shown in Fig. 2, spectrum B and differed significantly from the ^1^H spectrum before DTT treatment (Fig. 2, spectrum A). Nevertheless, the ^1^H resonances could be easily assigned, including the M8B and M5A methyls, which shifted to 36.7 and 35.4 ppm, respectively, while retaining a constant population ratio of approximately 2:1.

### Structural effects of disulfide bridge formation in WT Ngb

Most well-resolved heme resonances could be assigned in both WT_red_ and WT_ox_ (see Fig. 2) using 2D nuclear Overhauser effect spectroscopy and total correlation spectroscopy experiments (data not shown) and on the basis of the assignment of the mouse [28] and human [30] Ngb. Heme resonances shifted slightly upon cysteine reduction, indicating that the loss of the disulfide bridge perturbs the paramagnetic contact and pseudocontact shifts that dominate heme chemical shifts. In the absence of experimental structures for the human WT Ngb, the chemical shift analysis suggests a significant structural rearrangement in the heme vicinity upon cysteine oxidation. Nevertheless, the very similar overall pattern of the heme ^1^H spectrum in the presence or absence of disulfide bridge is consistent with only a limited structural change for the heme. As an illustration, the conserved order and relative distance for heme methyls indicated mostly unaffected nodal plane orientation for the iron ligands His96 and His64 [47, 48].

### NMR characterization of C120S and C46G/C55S/C120S Ngb mutants

Ngb contains three cysteines, and Cys120 is suspected to form intermolecular bridges. To probe such a possibility, the C120S mutant was produced. The ^1^H NMR spectra of this mutant in oxidized and reduced states (Fig. 2, spectra C and D) were essentially indistinguishable from those of their WT counterparts. This observation confirmed that a cross-linked Ngb monomer, if it exists, does not significantly contribute to the NMR spectra of WT Ngb, consistent with the findings of mass spectrometry. In addition, this demonstrated the intact heme environment upon mutation in line with the location of Cys120 in the remote GH loop, 17 Å from the heme. To understand how chemical modifications in the CD loop may affect protein function, we also produced the cysteine-free triple mutant (C46G/C55S/C120S). The crystal structure of this mutant is available [22], and despite a recent report on WT Ngb crystallography [49], it is still the sole experimental structure available for human Ngb. As visible in Fig. 2, spectrum E, the ^1^H NMR spectrum of triple-mutant Ngb was globally very similar to the spectra of other Ngb sequences, indicating a conserved global fold in triple-mutant Ngb. Notably, the closest similarity was observed with WT_red_ and reduced C120S Ngb, in line with the absence of a disulfide bridge in these species. Nevertheless, resolved heme resonances in triple-mutant Ngb slightly shifted with respect to those in WT_red_, indicating significant but limited reorganization in the heme vicinity upon mutations in the CD loop. As a conclusion, chemical modifications in the CD–D region bearing Cys46 and Cys55, such as oxidation of cysteines or amino acid substitution, consistently modify the heme environment. In contrast, the heme environment and structure are not sensitive to amino acid substitution in the GH loop bearing Cys120.

### Kinetics of cyanide binding to WT_ox_

**Fig. 3:**
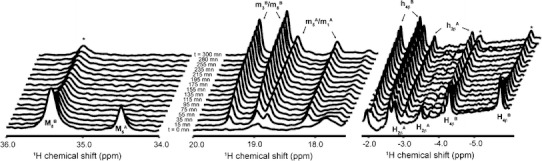
Time evolution of the 1D ^1^H spectrum of a 100 μM WT_ox_ sample after injection at *t* = 0 min of 500 μM KCN. Only well-resolved regions of the spectra are shown. The characteristic peaks of metNgb and cyanometNgb are labeled with the corresponding heme atoms using uppercase letters (*M*, *H*) and lowercase letters (*m*, *h*), respectively. Peaks labeled with a *star* correspond to metNgb that remains unbound at the end of the reaction

**Fig. 4:**
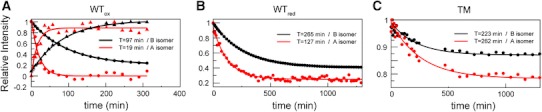
Time evolution of peak intensities during the reaction of cyanide binding to the metNgb forms WT_ox_ (**a**), WT_red_ (**b**), and TM (**c**). The time-dependent (decreasing) intensities for the M_8_^B^ (*black circles*) and M_5_^A^ (*red circles*) methyl resonances are scaled relative to the initial intensity (*t* = 0 min). In addition, the (increasing) intensities for the m_3_^B^/m_8_^B^ (*black triangles*) and h_2β_^A^ (*red triangles*) resonances in cyanometNgb are also reported for WT_ox_ in **a**. The *red curves* and the *black curves* represent the best-fit exponential curves for the experimental data obtained on the A isomer and the B isomer, respectively, and the resulting time constants *T* are also indicated for the two conformations

The cyanide anion CN^−^ is a known Ngb ligand. To obtain deep insight into the thermodynamics and kinetics of the interaction, we added 500 μM KCN to a solution of 100 μM Ngb with oxidized cysteines, the typical concentration of Ngb in retina cells. The binding kinetics was followed by real-time ^1^H NMR spectra collected at regular intervals. Typical spectra are shown in Fig. 3 for WT_ox_. The first spectrum after KCN injection (*t* = 15 min) was very similar to the reference spectrum (*t* = 0 min) collected in the absence of KCN despite smaller intensities for heme resonances. Over the course of the experiment, the intensity of the signals in the 34–36-ppm region gradually decreased until the intensity reached zero for M5A methyl and a plateau for M8B methyl (see Figs. 3, 4a). At the same time, the 18–20 ppm region, which is largely free of heme resonances in WT_ox_, displays new lines of growing intensities that reached a plateau at longer times after addition of KCN. The global spectral behavior was attributed to the remarkably slow binding of the CN^−^ ion to the heme Fe^3+^ associated with the high-spin to low-spin transition, resulting in a reduced paramagnetic shift contribution. The time course of the M5A and M8B peak intensities is reported in Fig. 4a (circles) and could be easily fitted to monoexponential functions with time constants of 19 and 97 min, respectively. The resonances corresponding to the CN^−^-bound Ngb showed reduced chemical shift dispersion and only a few isolated peaks showed high-quality intensity buildup. The time courses of two of them (h_2β_^A^ and m_3_^B^/m_8_^B^ protons) are also shown in Fig. 4a (triangles) and had very different time constants, which permitted assignment of the peaks to the cyanometNgb A isomer or the cyanometNgb B isomer. In particular, the CN^−^ binding reaction was completed for the A isomer 60 min after KCN injection as judged from the constant peak intensities for the metNgb and cyanometNgb signals after 1 h incubation (red curves in Fig. 4a). In contrast, the reaction with the B isomer was hardly finished after 5 h (black curves in Fig. 4a). The cyanometNgb signals were assigned to the corresponding atoms on the basis of a previous NMR study of mouse cyanometNgb [28, 29] and a pair of 2D total correlation spectroscopy/nuclear Overhauser effect spectroscopy spectra collected at the end of the reaction.

The second-order binding rate constants, *k*_obs_, were calculated for the A and B forms on the basis of the cyanide concentration and the time constants. This is possible because in this KCN concentration range, *k*_obs_ is linear with respect to cyanide concentration [12]. The values obtained are reported in Table 1 and strongly concur with the findings of a previous UV–visible study of cyanide binding to human Ngb [12]. This study revealed a biphasic binding mechanism with fast (1.65 M^−1^ s^−1^) and slow (0.37 M^−1^ s^−1^) binding rates, which could not be assigned to a specific molecular mechanism. The very similar on-rate values measured by NMR and UV–visible techniques demonstrated that the same phenomena were observed involving fast cyanide binding to the A isomer and slow binding to the B isomer, with about fivefold faster CN^−^ binding to the A isomer compared with the B isomer in the presence of an internal disulfide bridge. At the end of the binding reaction, the B isomer to A isomer population ratio was estimated to be conserved (about 2:1) on the basis of the relative peak intensities of the m_3_^B^/m_8_^B^ and m_1_^A^/m_5_^A^ methyl groups (Fig. 3).

**Table 1:** Association rate constants for binding of cyanide to neuroglobin (Ngb)

	*T* (min)	*k*_obs_ (M^−1^ s^−1^)
WT_ox_ (A isomer)	19	1.75
WT_ox_ (B isomer)	97	0.34
WT_red_ (A isomer)	127	0.26 (*K*_d_ = 153 μM)
WT_red_ (B isomer)	265	0.12 (*K*_d_ = 316 μM)
C120S_ox_ (A isomer)	18	1.85
C120S_ox_ (B isomer)	119	0.28
C120S_red_ (A isomer)	138	0.24
C120S_red_ (B isomer)	255	0.13
TM (A isomer)	262	0.12 (*K*_d_ = 2 mM)
TM (B isomer)	223	0.14 (*K*_d_ = 5 mM)

The time constants (*T*) as obtained from the exponential fit to the experimental intensities shown in Fig. 4 are reported for every Ngb state. The second-order rate constants (*k*_obs_) were calculated from the time constant and cyanide concentration (500 μM) assuming a linear relationship (1/*T* = *k*_obs_[CN^−^]). When determined, the dissociation constants (*K*_d_) calculated on the basis of the concentrations of the free and bound species at equilibrium are also reported.

*WT*_*ox*_ oxidized wild-type Ngb, *WT*_*red*_ reduced wild-type Ngb, *C120S*_*ox*_ oxidized C120S mutant, *C120S*_*red*_ reduced C120S mutant, *TM* C46G/C55S/C120S triple mutant

### Decreased binding rates upon reduction of cysteines

To obtain more insight into the influence of the disulfide bridge on ligand binding kinetics in human Ngb, we also monitored the kinetics of cyanide binding to a cysteine-reduced Ngb (WT_red_) sample in the same conditions as for WT_ox_ (Fig. 4b). During this long cyanide binding experiment, the absence of reducing agent in solution such as DTT might result in air reoxidation of the disulfide bridge that may interfere with the binding process. The rate of air reoxidation was estimated to be about 60 h from a similar experiment where cyanide was omitted and by following the increasing peak intensity of the WT_ox_ form. We concluded that the kinetics represented in Fig. 4b unambiguously reported on cyanide binding to WT_red_.

As illustrated in Fig. 4b, both the A isomer and the B isomer showed very slow binding in WT_red_, with time constants of 127 and 265 min, respectively. In the absence of a disulfide bridge, the A isomer also bound cyanide at a faster rate than the B isomer. When compared with WT_ox_, the on-rate values decreased by a factor of 5 and 2.5 for the A isomer and the B isomer, respectively, upon cysteine reduction, suggesting that the disruption of the disulfide bridge globally decelerates cyanide ligation. At equilibrium, and despite the excess of CN^−^ anion in solution, neither of the isomers were saturated in cyanide, and about 42 % of the A isomer and 24 % of the B isomer remained unbound. Thus, reduced cysteines in Ngb are associated with decreased affinity for cyanide.

The dissociation constants *K*_d,A_ and *K*_d,B_ for dissociation of cyanide from the A isomer and the B isomer, respectively, were calculated to 153 and 316 μM, respectively, on the basis of the concentrations of the free and bound species at equilibrium. The ratio *K*_d,B_/*K*_d,A_ was then estimated to be 2.1, which is very similar to the *k*_obs_^A^/*k*_obs_^B^ ratio of on-rate values (Table 1), calculated to be 2.2. This analysis demonstrated the very similar off-rate values for both complexes, estimated to be 3.9 × 10^−5^ s^−1^. The increased affinity of the A conformer versus the B conformer might then be explained by a faster on rate in the A isomer, suggesting easier access to heme iron or enhanced reactivity of the heme iron in this conformation.

### Cyanide binding to triple-mutant Ngb and C120S Ngb

Cyanide binding experiments were also conducted on the C120S mutant in the cysteine oxidized and reduced states. The time constants are reported in Table 1 and were almost identical to the values measured on the WT protein. As a consequence, the replacement of a sulfur atom by an oxygen atom at position 120 in the Ngb sequence has a negligible effect on the kinetics and thermodynamics of cyanide binding to Ngb, whether the internal disulfide bridge is formed or not. This observation parallels the similar heme environment in WT and C120S forms.

The analysis of ^1^H NMR spectra of the triple mutant suggested detectable conformational changes upon C46G/C55S double mutation near the heme pocket. To assess the impact of the mutation on the functional properties of the protein, we also monitored the reaction of potassium cyanide binding to triple-mutant Ngb (see Fig. 4c). In contrast to WT Ngb, quite similar binding time constants were obtained for the A isomer and the B isomer (223 and 262 min, respectively). The similar binding time constants for the A conformer and the B conformer in the triple mutant suggest that heme orientation had less impact on cyanide binding in this mutant. More importantly, and in line with the slower on rate, the affinity of triple-mutant Ngb for CN^−^ was significantly reduced as judged from the incomplete binding at equilibrium (90 and 80 % of free B isomer and free A isomer, respectively, at the end of the reaction). *K*_d_ and *k*_off_ were evaluated to be 2.0 mM and 2.5 × 10^−4^ s^−1^, respectively, for the A isomer and 5.0 mM and 6.9 × 10^−4^ s^−1^, respectively, for the B isomer. Since the properties of cyanide binding to WT Ngb and the C120S mutant were highly similar, this analysis pointed to the major impact of C46G/C55S mutations in the CD loop on the cyanide binding kinetics and thermodynamics of Ngb.

### Internal flexibility in the CD loop probed by MD

**Fig. 5:**
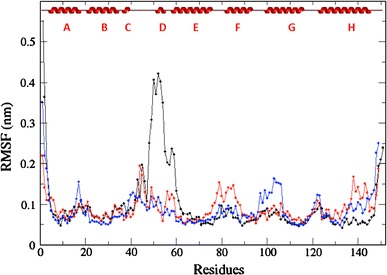
The root mean square fluctuation (*RMSF*) values calculated from the 2–12-ns molecular dynamics trajectories performed on WT_ox_ (*blue*), WT_red_ (*red*), and TM (*black*) Ngb are shown versus the protein sequence. The secondary structure elements are also shown

The previous observations shed light on the influence of the chemical composition of the CD region on the affinity of the Ngb ferric iron for CN^−^. To gain more detailed understanding, we analyzed molecular simulations performed on the different species. A 12-ns MD trajectory was produced for the triple-mutant Ngb state for comparison with recent simulations reported on WT_ox_ and WT_red_ [27]. All simulations were conducted on hexacoordinated bishistidine-liganded iron. Such conformations mimic the stable protein state in the absence of exogenous ligand and may contribute to an explanation of the mechanism underlying the slow ligand binding rate and relative accessibility of iron. The per-residue root mean square fluctuation (RMSF) was calculated for the MD simulations using principal component analysis and is shown for the three protein states in Fig. 5. The RMSF aims to represent the spatial extension of a given amino acid in the course of the trajectory, and is a good indicator of internal flexibility. As already noted [27], the formation of the disulfide bridge is associated with a slight decrease in flexibility in the CD and EF loops and increased flexibility in the AB and FG loops. The change in flexibility profile is likely due to the cross-link between the nine-residue distant cysteines Cys46 and Cys55, which strongly restrains atomic motions in the CD loop. For comparison, we also report the flexibility profile for the C46G/C55S/C120S triple mutant (black). We observed a dramatic modification in the two-cysteine (Cys46/Cys55) mutation region. The stretch displaying the highest RMSF in the triple-mutant Ngb simulation included Asn44-Glu60 that encompass the CD loop (Gln43-Ser50) and the following D helix (Ser51-Leu56). The highest RMSF values were obtained for Phe49 (4.3 Å) and Pro52 at the end of the CD loop. In contrast, the RMSF values in this region were barely higher than protein-averaged values in WT_red_, although a flexibility peak (at 2 Å) was visible close to Gln43. The higher motional amplitude upon mutation can be mostly explained by the replacement of a cysteine by a glycine residue at position 46 at the top of CD loop. The absence of a side chain in glycine is known to facilitate conformational transitions and is likely responsible for the increased motional amplitude in the CD loop of triple-mutant Ngb. The main conclusion drawn from the MD simulations is the gradual increase in conformational flexibility in the CD loop with the reduction of the internal disulfide bridge and with the mutation of cysteine residues in Ngb.

## Discussion

In this work, we assigned and analyzed most of the well-resolved heme NMR resonances of human WT Ngb in four stable states characterized by distinct heme orientations and cysteine oxidation states. To date no high-resolution NMR or crystal structure is available for human WT Ngb. The only human Ngb derived structure was obtained on the triple-cysteine-depleted mutant [22], and we also analyzed this triple mutant under the same sample conditions as the WT protein. The resonance assignment was greatly facilitated by the very similar heme ^1^H chemical shift pattern in all spectra, indicating that the heme environment is essentially conserved and that the WT protein and the triple mutant globally adopt similar structures. The population ratio of the A isomer and the B isomer, which is a sensitive probe of the local heme environment, was comparable in all protein states (WT_ox_/WT_red_/triple mutant) and further reinforces the impression of a conserved heme environment.

The Cys46–Cys55 distance predicted from the structure of triple-mutant Ngb is not compatible with easy S–S bond creation, and the CD–D region requires a conformational transition to accommodate the new bond. This structural change may in turn be transmitted to the heme crevice as a slight rearrangement, such as heme reorientation or rotation of the *g* tensor. Indeed, subtle conformational differences in the heme vicinity could be detected on the basis of heme ^1^H chemical shift variations upon cysteine oxidization/reduction or depletion. Such a conclusion is also supported by recent EPR [50] and MD studies, and the Phe28 (B10) residue was proposed to mediate the communication between the CD–D region and the heme [51]. There is now accumulating experimental and computational evidence that the dynamics of the CD–D region is profoundly enhanced upon disruption of the internal disulfide bond in WT Ngb [27, 52], suggesting that the cysteine redox state of the protein may influence protein function by modulating the flexibility and the conformation of the CD–D region. In this work we also found that the mutation of cysteine residues in the CD–D region resulted in heme displacement compared with WT_red_, in agreement with a previous Fourier transform IR study [52], further confirming the strong connection between the CD loop and heme conformation. Unfortunately, the absence of a high-resolution structure of WT Ngb excludes a more detailed description of the transition. We also demonstrated using MD simulations that the CD loop becomes even more flexible upon mutation of the cysteine residues to glycine or serine, which is likely due to the enhanced flexibility of glycine introduced at position 46. Of interest, mouse and human Ngb share high sequence identity (94 %), with an identical sequence in the CD loop except for position 46, which is occupied by a glycine in the mouse sequence and a cysteine in the human sequence. It is then expected that enhanced mobility in the CD loop might contribute to possible distinct functional properties for mouse and human Ngb.

Cyanide binding to metNgb (Fe^3+^) confirmed the extraordinarily slow kinetics of cyanometNgb formation that occurs on the hour timescale, and facilitated the NMR time-resolved study of the binding process. Mammalian myoglobins and hemoglobins are known to have relatively slow reactivity towards cyanide, with second-order rate constants on the order of 10^2^ M^−1^ s^−1^, but still two to three orders of magnitude faster than for Ngb. Two exceptions are the monomeric *Glycera dibranchiata* hemoglobin [53] and the H117A mutant of the truncated hemoglobin from the cyanobacterium *Synechocystis* sp. PCC 6803 [54], which have similar *k*_obs_ values compared with Ngb. Extensive studies performed on metmyoglobin [55–57] revealed the key parameters controlling cyanide reactivity and identified the critical role of residues at positions CD4, B10, E7, and E11 in controlling conditions for ligation in the distal pocket. Owing to its high p*K*_a_ (about 9), cyanide exists predominantly as HCN at near-neutral or slightly basic pH. HCN is the diffusive species carrying the cyanide anion, and after ionization, CN^−^ anions are released in the vicinity of Fe^3+^ for complexation. In the case of pentacoordinate heme, the entire binding process was proposed [55–57] to be governed by the strength of the water–Fe^3+^ coordination that needs to be fractured, by the dissociation rate of the HCN molecule within the protein, and by the steric hindrance and electrostatic interactions in the vicinity of the binding site of the external ligand. Obviously, for hexacoordinate heme, this model has to be adapted and the water–Fe^3+^ interaction has to be replaced by the distal histidine–iron contact. In *G. dibranchiata* hemoglobin, the heme is pentacoordinated, an atypical distal leucine residue replaces the widely conserved (E7) histidine (His64 in human Ngb), and an unusual phenylalanine residue is found at the B10 position, which is commonly occupied by a leucine residue in globins. The complete absence of amino acids having a polar or hydrogen-bonding acceptor/donor side chain contributes to the strong hydrophobicity of the distal heme pocket, which is an adverse factor for HCN ionization. In the case of *G. dibranchiata* hemoglobin, the absence of the histidine at the distal position was also proposed to explain the anomalous cyanide binding [58] because the replacement of histidine (E7) by hydrophobic residues considerably slowed the binding on-rate. This proposal does not hold for Ngb since the E7 position is occupied by the conserved His64 and an alternative mechanism must be invoked. For hexacoordinate heme, the distal histidine replaces a water molecule as the sixth iron ligand and exogenous ligand must displace this endogenous ligand prior to ligation. The slow histidine dissociation appears as an additional tool to regulate globin ligation and the internal competitive ligation can be described by Eq. 1 [59]:

1 $$ {\text{Ngb}}_{\text{hexa}} \mathop{\rightleftarrows}\limits^{{k_{\text{{-}H}} }}_{{k_{\text{H}} }}{\text{Ngb}}_{\text{penta}} \mathop{\longrightarrow}\limits^{{k_{{{\text{on}},{\text{L}}}} \left[ L \right]}}{\text{Ngb}}_{\text{penta}} L. $$

In the case of Ngb, and for the ligands tested so far (CN^−^, NO, CO, O_2_) [12, 24, 59, 60], the complex formation rate constant *k*_obs_ has been found to be proportional to ligand concentration, in agreement with the rapid equilibration of His64–iron association and dissociation prior to ligation, i.e., *k*_on,L_ ≪ *k*_H_, *k*_−H_. Under such circumstances, *k*_obs_ can be approximated to *k*_on,L_/(1 + *K*_H_), with *K*_H_ = *k*_H_/*k*_−H_, and therefore the binding kinetics is directly governed by histidine–iron bond stability (*K*_H_) and the ligand diffusion rate (*k*_on,L_). Irrespective of the ligand used, the binding kinetics of Ngb reveals a biphasic nature with a slow and a fast component. Such an observation can be attributed to distinct Ngb substates having different binding properties. However, the highly heterogeneous nature of Ngb severely hindered the unambiguous assignment of the binding kinetics to the corresponding protein state in the past. In the simpler situation of mouse Ngb, which cannot form an internal disulfide bridge, the NMR technique was used to demonstrate that the heme orientation is directly responsible for a twofold difference in cyanide association rate constant [28]. In the present study conducted on human Ngb, we detected and unambiguously assigned NMR signals and monitored the cyanide binding rate constants even in a heterogeneous protein background. Regardless of the cysteine oxidation state, the A conformation was found to bind cyanide faster than the B conformation. Our study has therefore greatly facilitated the unambiguous assignment of the fast (and slow) phase of cyanide binding to the A (and B, respectively) heme orientation from the previous UV–visible study [12].

Our understanding of how heme disorder affects ligand reactivity can now be greatly improved. As seen before, the apparent association rate (*k*_obs_) depends on the efficiency of the diffusion of the ligand towards the pentacoordinated heme iron (*k*_on,L_) and on the His64–iron bond stability (*K*_H_). In principle, heme heterogeneity may affect both parameters. Nevertheless, since the heme controls the precise position of the heme iron atom, it is expected that heme primarily governs the histidine–iron interaction, which was initially proposed to explain the heme-orientation-dependent ligation rate for mouse Ngb [28]. Convincingly, different *k*_−H_ values were extracted from the fast and slow phases of CO ligand binding to Ngb, whereas similar *k*_on,L_ values were fitted for the two phases [59], suggesting that heme orientation essentially conditions the iron–histidine bond stability. In mouse Ngb, the dissociation of His64 from iron has been associated with heme sliding towards the bottom of the heme cavity, while keeping the His64 side chain at the same position [26]. It is then likely that the energy barrier corresponding to heme sliding is lowered in the case of the A conformer, thus accelerating the histidine–iron bond rupture. For example, the steric hindrance in the vicinity of the Phe106 side chain, which exhibits the largest conformational change upon heme sliding, may play a significant role in controlling the structural transition. Indeed, this region is less crowded in the A conformer than in the B conformer.

A marked deceleration in cyanide complex formation was observed upon reduction of the disulfide bridge, which in turn was associated with reduced affinity. A similar observation was also reported for O_2_ and CO ligands [32, 34]. Therefore, faster ligand binding, and increased affinity for the ligand, appears to be a general hallmark of Ngb containing an internal disulfide bridge, irrespective of the type of ligand. The histidine–iron bond was measured to be stabler in the absence of a disulfide bridge [32, 61], which may explain the greater difficulty for the ligand to access iron in this state. A theoretical work further supported the observation that the pentacoordinate form was stabilized by the existence of the disulfide bridge [35]. Therefore, the stabler histidine–iron bond in the absence of an internal disulfide bridge might explain the slower reactivity. Notably, the ratio of A/B binding rates also significantly decreases from 5 to 2 upon cysteine reduction, which may indicate reequilibration of histidine dissociation rates between the two conformations in the reduced Ngb. This trend is further confirmed by the similar on-rate values for the two heme orientations in the triple mutant. An increase in backbone flexibility was found in the CD–D region upon cysteine reduction and was even greater upon C46G/C55S mutations (MD study), which suggests that elevated flexibility in this region may contribute to stabilize the His64–Fe^3+^ bond. This analysis further indicates that the triple-mutant Ngb poorly mimics the function of the WT protein. This may be attributed to the small structural changes caused by the mutations as revealed from the heme chemical shift analysis. This reinforces the need for an experimental structure of the native protein.

**Fig. 6:**
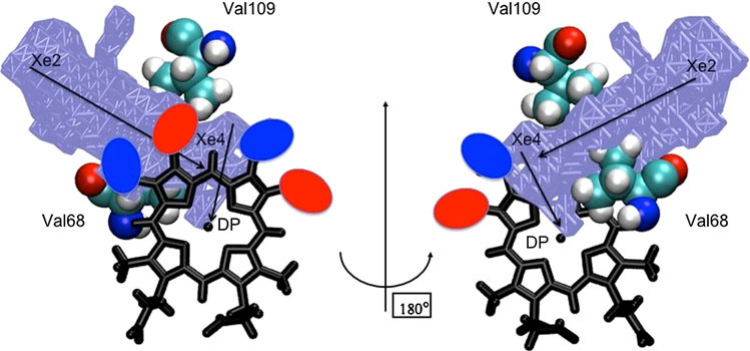
Close-up schematic view of the heme region of Ngb. The heme skeleton is represented as *black sticks*. The mechanically sensitive Val68 (E11) and Val109 (G8) residues are represented as *cyan spheres* and the Xe2/Xe4 cavities and the distal pocket (*DP*) are indicated as an *ice blue surface*. In this scheme, the *red* and *blue ovals* indicate the volumes occupied by the vinyl groups of the A and B heme conformers, respectively. Consequently, the *red ovals* and *blue ovals* indicate the volumes occupied by methyl groups of the B and A heme conformers, respectively. The view on the *right* was obtained from a 180° rotation of the same scheme around the vertical axis

Whether the rate of cyanide binding to the pentacoordinate Ngb is heme-conformation-dependent is not clear yet. This reaction depends on multiple elementary events, including ligand migration in solvent and in the protein matrix and ionization of HCN to H^+^ and CN^−^. An internal cavity network exists in the globular matrix of globins that is considered as a possible pathway for ligand migration towards the iron site [62–73]. The role of internal cavities may even be more important for hexacoordinated proteins, such as Ngb, because the histidine–iron coordination blocks the natural ligation pathway observed in hemoglobin and myoglobin. Therefore, small diatomic molecules are thought to rapidly diffuse within the internal network of Ngb prior to ligation and the ensemble of temporary docking sites may then play the role of a reservoir to facilitate access to the final and stabler intermolecular complex [74]. Such a mechanism may also explain why the rate of binding to the pentacoordinate state is several orders of magnitude slower (estimated here to be *k*_on,CN_ = 6.7 × 10^3^ M^−1^ s^−1^) than the rate based on diffusion-control theory (*k*_on,CN_ = 10^8^–10^9^ M^−1^ s^−1^). It has recently been proposed that this migration of the ligand inside the internal globin cavities can be controlled by four highly conserved residues which are located at the cavity frontiers [75]. As shown in Fig. 6, the Val109 and Val68 residues, which are mechanically sensitive residues that control ligand diffusion between the Xe2 and Xe4 docking sites [27], are relatively close to the heme. The close proximity between these rigid residues and the prosthetic group may potentially induce a blocking for the heme displacement inside the cavities which might contribute to our understanding of the extremely slow diffusion of ligands towards the Ngb heme when compared with other globins (see Fig. 6). Since the B heme conformer can be formally obtained from the A conformation by exchanging the position of methyl and vinyl groups, we represented in Fig. 6 the heme-orientation-dependent vinyl location as colored ovals. It is apparent from this figure that the location of the relative positions of the vinyl and methyl groups, i.e., the heme orientation, may have a significant impact on the ligand diffusion between cavities Xe2 and Xe4 towards the distal pocket through the steric effects induced by the different size for the methyl and vinyl groups. This provides a rationale for the heme-orientation-dependent ligand diffusion properties.

Although the precise function of Ngb in the cell is not yet fully understood, there is now a wealth of experimental data demonstrating the redox-dependent ligand binding properties of Ngb in vitro. This is illustrated here by the threefold to sevenfold reduction in cyanide ligation rate for Ngb upon cysteine reduction. The redox-dependent activity was proposed to explain the role of Ngb in protecting neuronal cells against oxidative stress induced under conditions such as hypoxia, ischemia, and stroke [13, 16, 17, 76]. However, Ngb displays large heterogeneity in binding properties owing to heme disorder and, for example, the B conformation with an internal disulfide bridge and the A conformation with reduced cysteines have similar ligand reactivity. Therefore, although from the macroscopic point of view Ngb experiences lower averaged reactivity in reducing conditions, the distributions of activity for the oxidized and reduced Ngb partially overlap owing to heme disorder, which sheds light on the requirement of a better description of the behavior of Ngb at the microscopic level. Alternatively, since heme orientation modulates Ngb activity, it may play a yet unrecognized role in regulating its function.
